# Waist to height ratio is associated with an increased risk of mortality in Chinese patients with heart failure with preserved ejection fraction

**DOI:** 10.1186/s12872-021-02080-9

**Published:** 2021-05-28

**Authors:** Jianqiao Chen, Man Li, Benchuan Hao, Yulun Cai, Huiying Li, Wenli Zhou, Yujian Song, Shiqi Wang, Hongbin Liu

**Affiliations:** 1 Geriatric Cardiology Department of The Second Medical Center & National Clinical Research Center for Geriatric Diseases, Chinese PLA General Hospital, #28 Fuxing Road, Beijing, 100853 China; 2 General Department of Zhengzhou First People’s Hospital, #56 Dong Dajie, Guancheng Hui District, Zhengzhou City, 450000 Henan Province China

**Keywords:** Abdominal obesity, Waist to height ratio, Prognosis, Heart failure with preserved ejection fraction

## Abstract

**Background:**

Abdominal obesity as a predominant comorbidity has played a key role in the incidence and worsening of heart failure with preserved ejection fraction (HFpEF), and waist-to-height ratio (WHtR) behaves better than waist circumference or body mass index in evaluating abdominal obesity. While the association between WHtR and all-cause death in Chinese patients with HFpEF remains unclear.

**Methods:**

Patients with stable HFpEF (N = 2041) who presented to our hospital from January 2008 to July 2019 were divided into low-WHtR (< 0.5, N = 378) and high-WHtR (≥ 0.5, N = 1663). Multivariable Cox proportional-hazard models were used to examine the association of WHtR with all-cause death.

**Results:**

The average age was 76.63 ± 11.44 years, and the mean follow-up was 4.53 years. During follow-up, 185 patients (9.06%) reached the primary outcome of all-cause death. As for the secondary outcome, 79 patients (3.87%) experienced cardiovascular death, 106 (5.19%) had non-cardiovascular death, and 94 (4.61%) had heart failure rehospitalization. After multivariable adjustment, a higher WHtR was significantly associated with the increased risks of all-cause death [adjusted hazard ratios (HR) 1.91, 95% confidence interval (CI) 1.06–3.45, *p* = 0.032], cardiovascular death (adjusted HR 2.58; 95% CI 1.01–6.67, *p* = 0.048), and HF rehospitalization (adjusted HR 3.04; 95% CI 1.26–7.31, *p* = 0.013).

**Conclusions:**

Higher WHtR is an independent risk factor for all-cause death in Chinese patients with HFpEF.

**Supplementary Information:**

The online version contains supplementary material available at 10.1186/s12872-021-02080-9.

## Introduction

Heart failure with preserved ejection fraction (HFpEF) contributes to nearly half of all heart failure (HF) cases, and this proportion has been increasing in recent years [1, 2]. Its significant phenotypic heterogeneity from fundamental pathophysiology, which is extremely complicated and poorly understood, creates the primary obstacle to therapy. Recent reports have proposed that a systemic proinflammatory state driven by multiple comorbidities, which can cause myocardial inflammation, oxidative stress, and fibrosis, may also play a significant role in HFpEF development [2–4]. Additionally, changes in cardiomyocyte signaling pathways accelerate cardiomyocyte remodeling and microvascular dysfunction, eventually leading to diastolic dysfunction [3].

Abdominal adipose deposition has a close relationship with systemic inflammation [5] and is also a prominent feature of HFpEF [6, 7]. Several studies have demonstrated that abdominal obesity may predict adverse consequences and a greater risk of all-cause mortality in patients with HFpEF [8, 9]. However, existing evaluation indicators such as body mass index (BMI) evaluate systemic obesity but fail to evaluate the fat or muscle tissue proportion in the body [10]. In addition, genetic and environmental factors may also complicate the relationship between BMI and the body fat rate and distribution among different ethnic groups [11]. Although waist circumference (WC) can reflect abdominal obesity, this measure may not identify obesity in individuals with shorter height, normal WC and more body fat. As for waist-to-hip ratio, gender and age differences make it unsuitable for accurate reflection of abdominal fat changes.

Waist to height ratio (WHtR) is a concept proposed to improve upon waist-to-hip ratio. When evaluating abdominal obesity, WHtR balances the effect of height on the basis of WC and overcomes the disadvantage of waist-to-hip ratio, which has a different reference value for each sex. A previous study showed that for Asian populations with low BMI, WHtR was more suitable than WC for exploring associations between obesity and cardiovascular (CV) disease [12]. However, little is known about the association of WHtR with outcomes in Chinese patients with HFpEF.

## Methods

### Patients

This study was approved by the Ethics Board of the Chinese PLA General Hospital and was conducted in line with the ethical guidelines of the 1975 Declaration of Helsinki. Written informed consent was obtained from each patient. The datasets used and/or analyzed during the current study are available from the corresponding author on reasonable request.

This prospective study recruited 3623 community dwelling HFpEF patients who received physical examination at the Chinese PLA General Hospital (Beijing, China) from January 2008 to July 2019. Eligible HFpEF patients had a history of HF hospitalization and were stable and well-compensated without medication changes for at least 6 weeks prior to enrollment. Referring to the 2016 European Society of Cardiology guidelines [13], the diagnosis of HFpEF should satisfy the following criteria: patients with (1) HF syndromes and/or signs, (2) left ventricular ejection fraction (LVEF) > 50%, (3) N-terminal pro-brain natriuretic peptide (NT-proBNP) > 125 pg/ml in sinus rhythm and > 375 pg/ml in atrial fibrillation, and (4) evidence of left atrium enlargement and/or left ventricle hypertrophy or diastolic dysfunction, which was identified by echocardiography. Patients who had one of the following conditions were excluded: severe valvular disease, hospitalization for uncompensated HF or unstable coronary heart disease in the prior 6 weeks, heart transplant, chronic kidney disease of stage 4 or 5, severe liver disease (aspartate aminotransferase and alanine aminotransferase levels > 3.0 times the upper limit of the normal range in the local laboratory) or receiving palliative care. We collected patient detailed medical history, baseline clinical characteristics, laboratory indexes and echocardiographic parameters on the day of the physical examination. For this study, patients with missing information regarding abdominal obesity and adjusted factors were excluded (N = 1582), leading to a final sample of 2041.

### Definitions

WHtR, defined as WC divided by height, was evaluated as a categorical variable. In each patient, height was measured without shoes, and WC was measured at the level of umbilicus, with the tape snugly positioned on but not compressing the skin. On the basis of a previous study in a Chinese population [14] and a meta-analysis [15], patients were divided into low-WHtR group (WHtR < 0.5) and high-WHtR group (WHtR ≥ 0.5).

For non-smokers or non-drinkers, they were defined as individuals who never had this behavior or quitted more than 1 year. Smokers or drinkers included those currently had this behavior and those who quitted less than 1 year before enrollment.

Referring to the Charlson Comorbidity Index (CCI) [16], the number of patient comorbidities was used to evaluate the severity of patient comorbidities. For the present study, we included the comorbidities with 1 point in CCI (myocardial infarction, peripheral vascular disease, cerebrovascular disease, dementia, chronic lung disease, connective tissue disease, ulcer disease, mild liver disease and diabetes), hypertension and atrial fibrillation, and excluded the comorbidities scored ≥ 2 point in CCI, as these comorbidities (such as severe liver disease, metastatic tumors, leukemia, etc.) might confound the association between abdominal obesity and mortality. As our analysis was entirely among HFpEF patients, the 1 point added for HF was not included in the final number of comorbidities.

We used the biplane modified Simpson’s method to measure left ventricular end-diastolic volume (LVEDV) and end-systolic volume (LVESV) in the apical 4- and 2-chamber views, and then calculated LVEF as follows: (LVEDV—LVESV)/LVEDV. Relative wall thickness (RWT) and left ventricular mass index (LVMI) were used to evaluate left ventricular hypertrophy. The former was calculated as twice the thickness of the left ventricular posterior wall (LVPWT) divided by the left ventricular end-diastole diameter (LVEDD), and the latter was calculated as left ventricular mass (LVM) normalized to body surface area (calculated by formula of Stevenson). The following formula was used to calculate LVM: LVM (g) = 0.8 × 1.04 × [(interventricular septal thickness (IVS) + LVEDD + LVPWT)^3^ − (LVEDD)^3^] + 0.6 [17, 18].

### Follow-up and outcomes

Until December 31, 2020, all patients were followed up via telephone or medical record every 6 months for the primary outcome, which was defined as all-cause death. Furthermore, cardiovascular and non-cardiovascular death as well as HF rehospitalization were assessed as secondary outcomes respectively, in order to investigate the cause of death and the worsening of HF. Cardiovascular death referred to death from myocardial infarction, stroke, sudden death, heart failure and pulmonary embolism, while non-cardiovascular death included death from cancer, infection, traffic accidents and other non-cardiovascular event. For patients who did not have an event, survival time was defined as the period from the day of physical examination to the last date of follow-up. Patient outcomes during follow-up were adjudicated by two experienced cardiologists.

### Statistical analysis

Patient baseline characteristics are presented as frequencies (%) and mean ± standard deviation (SD) or median (interquartile range) for categorical and continuous variables, respectively. Differences between groups were evaluated by chi-square tests for categorical variables and Student’s *t* test for continuous variables. For continuous variables with non-normal distribution, that is, NT-proBNP, Mann–Whitney U test was used to compare differences between groups. The association of WHtR with outcome was evaluated using Kaplan–Meier survival curves and Cox proportional hazards models adjusted for the following covariates: In Model 1, age, gender, smoking and alcohol; and in Model 2, the variables in Model 1 plus systolic blood pressure (SBP), diastolic blood pressure (DBP), heart rate, BMI (calculated by weight divided by the square of height), WC, estimated glomerular filtration rate (eGFR, calculated by a modified Modification of Diet in Renal Disease), NT-proBNP, comorbidities, and the use of angiotensin-converting enzyme inhibitors (ACEI)/angiotensin receptor blockers (ARB), beta blockers, diuretics or statin medication. These relevant covariates were chosen because previous publications have identified them as significant independent risk factors of CV outcome [19]. The low-WHtR category was used as the reference group for comparison regarding the relationship between WHtR and predefined end points. In addition, we used Cox regression analysis adjusted by covariates in Model 2 to compare the prognostic impact among BMI (< 18.5 kg/m^2^, 18.5–23.9 kg/m^2^, 24–27.9 kg/m^2^ or ≥ 28 kg/m^2^), WHtR (< 0.5 or ≥ 0.5) and WC in patients with HFpEF.

Moreover, Kaplan–Meier survival curves were constructed to assess the outcomes in propensity score-match patients with low and high WHtR [20]. We used 1:1 propensity score matching, which was performed by a logistic regression model that regarded WHtR as the group indicator and the potential confounders as predictors: age, gender, smoking, alcohol, BMI, comorbidities, and the use of ACEI/ARB, beta blockers, diuretics and statin medication. Sensitivity analysis was further performed to explore the association between WHtR and all-cause death among the following subgroups: age (< 75 or ≥ 75 years), obesity (BMI < 28 kg/m^2^ or ≥ 28 kg/m^2^) and comorbidities (0–2 or ≥ 3). To explore effect modification, we tested for interactions between WHtR and these subgroups in multivariable model 2. SPSS 26.0 software (IBM Corporation, Armonk, NY, USA) was used to perform statistical analyses, and GraphPad Prism 9.0.0 software (San Diego, CA) was used for drafting figures. A *p* value of < 0.05 was considered significant.

## Results

### Baseline characteristics

A total of 2041 patients with HFpEF were included in this study, of whom 378 and 1663 HFpEF patients had low and high WHtR, respectively. Detailed baseline characteristics are shown in Table 1. Anthropometric parameters in HFpEF patients were significantly increased compared with the normal values [21–23]. The average WC and BMI were 91.91 ± 8.85 cm and 24.45 ± 3.11 kg/m^2^, respectively; 83.6% of patients had abdominal obesity (WC ≥ 85 cm in men or ≥ 80 in women), and 57.2% were overweight (BMI 24–27.9 kg/m^2^) or obese (BMI ≥ 28 kg/m^2^). Compared with patients with low WHtR, those with high WHtR showed significantly higher mean age, WC, BMI, SBP and the proportion of using ACEI/ARB, beta blocker and diuretic. There were no significant differences in the proportion of gender, smoking, alcohol and using statins, as well as DBP, fasting glucose or eGFR between the two groups. In addition, patients with high WHtR also had significantly higher levels of left ventricular inner diameters (IVS, LVPWT, LVEDD and LVESD), left ventricular volumes (LVESV and LVEDV), left atrial diameter, LVEF, LVMI, RWT and tricuspid regurgitation (TR) velocity. The average number of comorbidities was 2.38. Compared with patients with low WHtR, those in high WHtR had a significantly higher number of comorbidities (1.91 ± 1.45 vs. 2.49 ± 1.45, *p* < 0.001), as well as the higher prevalence of atrial fibrillation (10.05% vs. 15.39%, *p* = 0.004), ischemic heart disease (3.97% vs. 7.64%, *p* = 0.005), hypertension (48.41% vs. 70.35%, *p* < 0.001) and diabetes (26.98% vs. 35.96%, *p* < 0.001).

**Table 1:** Baseline characteristics by WHtR categories

Variable	Low-WHtR (N = 378)	High-WHtR (N = 1663)	All (N = 2041)	*p*-value
Age (yrs)	74.32 ± 11.90	77.15 ± 11.27	76.63 ± 11.44	< 0.001^†^
Male (%)	358 (94.71)	1598 (96.10)	1956 (95.84)	0.225
Smoking (%)	129 (34.13)	636 (38.52)	765 (37.70)	0.112
Alcohol (%)	114 (30.16)	533 (32.19)	647 (31.81)	0.445
WC (cm)	80.20 ± 5.29	94.57 ± 7.17	91.91 ± 8.85	< 0.001^†^
BMI (kg/m^2^)	22.40 ± 3.21	24.92 ± 2.89	24.45 ± 3.11	< 0.001^†^
SBP (mmHg)	129.78 ± 17.27	134.12 ± 16.90	133.32 ± 17.05	< 0.001^†^
DBP (mmHg)	70.64 ± 9.97	71.07 ± 10.10	70.99 ± 10.08	0.459
HR (bpm)	71.96 ± 10.63	72.03 ± 10.81	72.02 ± 10.77	0.912
FG (mmol/L)	5.70 ± 1.78	5.83 ± 1.69	5.80 ± 1.67	0.174
eGFR (mL/min per 1.73 m^2^)	90.03 ± 23.13	88.99 ± 25.04	89.18 ± 24.70	0.461
NT-proBNP (pg/mL)	373.15 (240.68–474.20)	494.60 (244.00–644.8)	390.30 (343.00–537.50)	0.016^†^
*Echocardiography*				
IVS (mm)	11.09 ± 1.10	12.46 ± 1.32	12.20 ± 1.29	< 0.001^†^
LVPWT (mm)	9.78 ± 0.93	10.17 ± 0.95	10.10 ± 0.96	< 0.001^†^
LVEDD (mm)	52.29 ± 3.10	54.25 ± 3.32	53.89 ± 3.30	< 0.001^†^
LVESD (mm)	39.85 ± 2.61	40.83 ± 3.07	40.65 ± 3.02	< 0.001^†^
LAD (mm)	34.34 ± 3.75	36.74 ± 4.09	36.30 ± 4.13	< 0.001^†^
LVESV (mL)	40.98 ± 8.71	44.24 ± 10.35	43.64 ± 10.14	< 0.001^†^
LVEDV (mL)	109.49 ± 16.53	115.51 ± 18.92	114.39 ± 18.64	< 0.001^†^
LVEF (%)	62.54 ± 4.29	61.53 ± 4.83	61.72 ± 4.75	< 0.001^†^
TR velocity (m/s)	3.24 ± 1.79	3.42 ± 0.36	3.39 ± 0.83	0.017^†^
LVMI (g/m^2^)	119.36 ± 17.53	135.74 ± 19.74	122.65 ± 19.42	< 0.001^†^
RWT	0.426 ± 0.042	0.434 ± 0.0419	0.428 ± 0.042	0.001^†^
*Medication (%)*				
ACEI/ARB	90 (23.81)	634 (38.12)	724 (35.47)	< 0.001^†^
Beta blocker	79 (20.90)	502 (30.19)	581 (28.47)	< 0.001^†^
Diuretic	33 (8.73)	212 (12.79)	245 (12.00)	0.030^†^
Statins	232 (61.38)	1055 (63.44)	1287 (63.06)	0.453
*Number of comorbidities*	1.91 ± 1.45	2.49 ± 1.45	2.38 ± 1.46	< 0.001^†^
0–2	271 (71.69)	874 (52.56)	1145 (56.10)	< 0.001^†^
3–4	83 (21.96)	638 (38.36)	721 (35.33)	< 0.001^†^
≥ 5	24 (6.35)	151 (9.08)	175 (8.57)	< 0.001^†^
*Medical history (%)*				
Atrial fibrillation	38 (10.05)	256 (15.39)	294 (14.40)	0.004^†^
Ischemic heart disease	15 (3.97)	127 (7.64)	142 (6.96)	0.005^†^
Hypertension	183 (48.41)	1170 (70.35)	1353 (66.29)	< 0.001^†^
Diabetes	102 (26.98)	598 (35.96)	700 (34.30)	< 0.001^†^

Data are presented as mean ± SD or as percentages

WHtR, indicates waist to height ratio; WC, waist circumference; BMI, body mass index; SBP, systolic blood pressure; DBP, diastolic blood pressure; HR, heart rate; FG, fasting glucose; eGFR, estimated glomerular filtration rate; NT-proBNP, N-terminal pro-brain natriuretic peptide; IVS, interventricular septal thickness; LVPWT, left ventricular posterior wall thickness; LVEDD, left ventricular end diastolic diameter; LVESD, left ventricular end systolic diameter; LAD, left atrial diameter; LVESV, left ventricular end systolic volume; LVEDV, left ventricular end diastolic volume; LVEF, left ventricular ejection fraction; TR, tricuspid regurgitation; LVMI, left ventricular mass index; RWT, relative wall thickness; ACEI, angiotensin-converting enzyme inhibitors; ARB, angiotensin receptor antagonist

Pairwise comparisons: ^†^significant for high-WHtR versus low-WHtR

### WHtR and outcomes

**Fig. 1:**
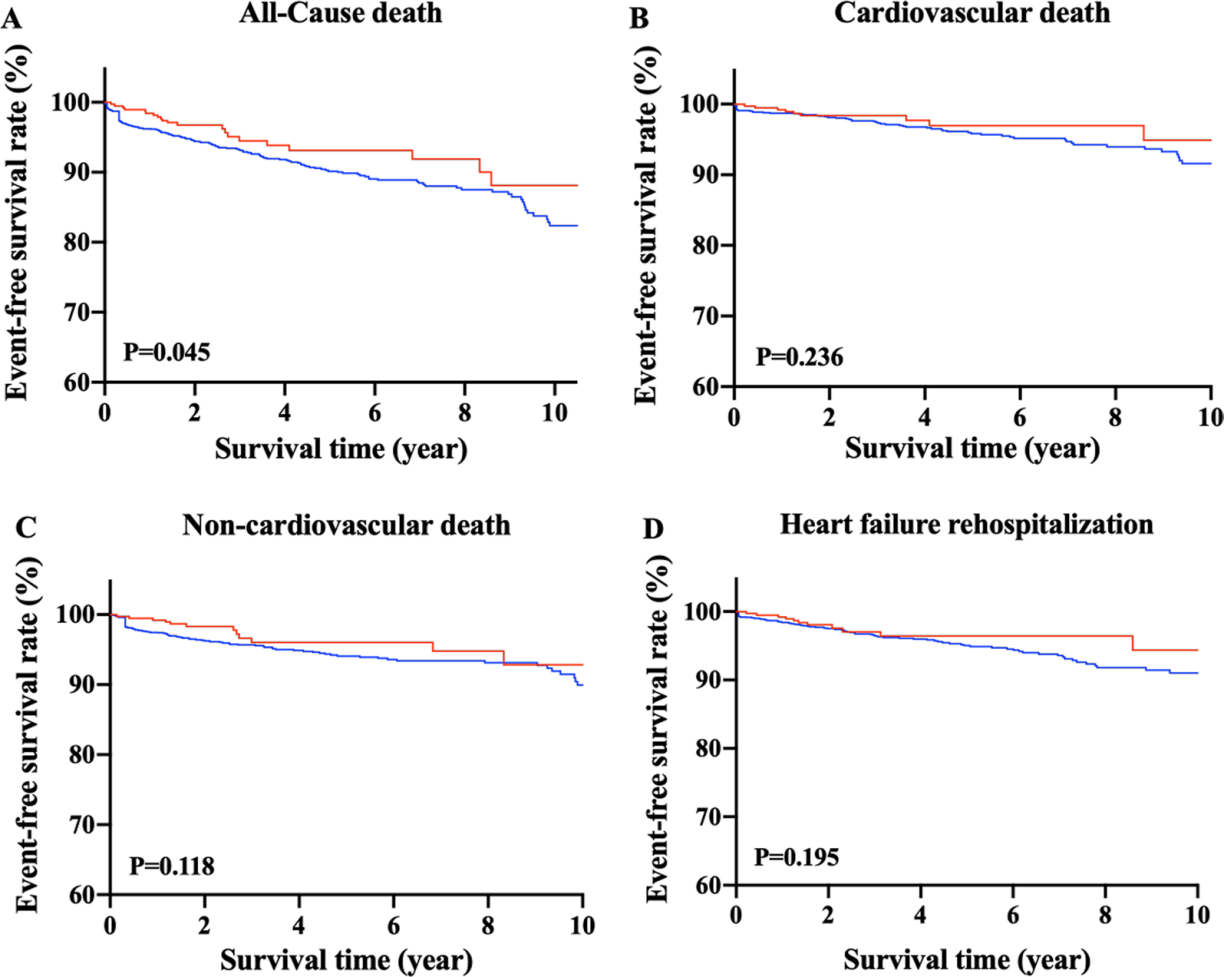
Kaplan–Meier survival curves for each outcome in heart failure with preserved heart failure patients with the low and high waist to height ratio. Event-free survival rates from (**A**) all-cause death, (**B**) cardiovascular death, (**C**) non-cardiovascular death, and (**D**) heart failure rehospitalization

The 2041 HFpEF patients were followed up for an average of 4.53 years, with no patients lost to follow-up. Among them, 185 (9.06%) experienced cardiovascular (N = 79, 3.87%) or non-cardiovascular death (N = 106, 5.19%), and 94 (4.61%) had HF rehospitalization. Kaplan–Meier survival curves and cumulative event rates for each predefined outcome in HFpEF patients by WHtR are shown in Fig. 1 and Table 2, respectively. The unadjusted risks of cardiovascular death (Fig. 1B), non-cardiovascular death (Fig. 1C) and HF rehospitalization (Fig. 1D) did not differ significantly between the two groups. The unadjusted risk of all-cause death (Fig. 1A) in the high-WHtR group was significantly higher than that in the low-WHtR group. After multivariable adjustment, hazard ratio (HR) for neither outcome showed statistical significance between the two groups in Model 1. However, after multivariate analysis in Model 2, higher WHtR was significantly associated with the increased risks of all-cause death (adjusted HR: 1.91, 95% confidence interval (CI): 1.06–3.45, *p* = 0.032), cardiovascular death (adjusted HR: 2.58; 95% CI: 1.01–6.67, *p* = 0.048) and HF rehospitalization (adjusted HR: 3.04; 95% CI: 1.26–7.31, *p* = 0.013), while WHtR was not associated with the risk of non-cardiovascular death (adjusted HR: 1.51; 95% CI: 0.71–3.22, *p* = 0.285). The comparison of the prognostic impact among BMI (< 18.5 kg/m^2^, 18.5–23.9 kg/m^2^, 24–27.9 kg/m^2^ or ≥ 28 kg/m^2^), WHtR (< 0.5 or ≥ 0.5) and WC showed that the association of both BMI and WC with each endpoint was not always statistically significant, indicating that WHtR seems to behave better in predicting the risk of adverse outcome in our patients with HFpEF (Table 3). In Additional file 1, we presented the HRs (95% CIs) of the covariates in Model 2. As we can see, age, DBP, number of comorbidities, eGFR and NT-proBNP were independent risk factors of all-cause death in HFpEF patients, with the HRs (95% CI) of 1.16 (1.13–1.19), 0.96 (0.95–0.98), 1.01 (1.00–1.02), 1.61 (1.11–2.03) and 1.29 (1.16–1.44), respectively.

**Table 2:** Outcome in HFpEF patients by WHtR category

	Low-WHtR (N = 378)	High-WHtR (N = 1663)	*p*-value
*All-cause death*			
N	21	164	
Event rate	5.56	9.86	
Unadjusted HR (95% CI)	1.00 (ref)	1.57(1.06–2.31)	0.045
Model 1: adjusted HR (95% CI)	1.00 (ref)	1.40(0.89–2.21)	0.151
Model 2: adjusted HR (95% CI)	1.00 (ref)	1.91(1.06–3.45)	0.032
*Cardiovascular death*			
N	9	70	
Event rate	2.38	4.21	
Unadjusted HR (95% CI)	1.00 (ref)	1.52(0.83–2.75)	0.236
Model 1: adjusted HR (95% CI)	1.00 (ref)	1.33(0.66–2.66)	0.429
Model 2: adjusted HR (95% CI)	1.00 (ref)	2.58(1.01–6.67)	0.048
*Non-cardiovascular death*			
N	12	94	
Event rate	3.18	5.65	
Unadjusted HR (95% CI)	1.00 (ref)	1.61(0.97–2.66)	0.118
Model 1: adjusted HR (95% CI)	1.00 (ref)	1.45(0.79–2.65)	0.232
Model 2: adjusted HR (95% CI)	1.00 (ref)	1.51(0.71–3.22)	0.285
*Heart failure rehospitalization*			
N	11	83	
Event rate	2.91	4.99	
Unadjusted HR (95% CI)	1.00 (ref)	1.51(0.88–2.60)	0.195
Model 1: adjusted HR (95% CI)	1.00 (ref)	1.29(0.69–2.43)	0.427
Model 2: adjusted HR (95% CI)	1.00 (ref)	3.04(1.26–7.31)	0.013

HFpEF, heart failure with preserved ejection fraction; WHtR, waist to height ratio; HR, hazard ratio; CI, confidence interval

**Table 3:** Comparison of prognosis value among WHtR, WC and BMI in Model 2

Covariates	All-cause death		Cardiovascular death		Non-cardiovascular death		Heart failure rehospitalization	
	HR (95% CI)	*p*	HR (95% CI)	*p*	HR (95% CI)	*p*	HR (95% CI)	*p*
*WHtR*								
< 0.5	1.00 (ref)		1.00 (ref)		1.00 (ref)		1.00 (ref)	
≥ 0.5	1.91 (1.06–3.45)	0.032	2.58 (1.01–6.67)	0.048	1.51 (0.71–3.22)	0.285	3.04 (1.26–7.31)	0.013
*BMI (kg/m*^*2*^*)*								
< 18.5	1.00 (ref)		1.00 (ref)		1.00 (ref)		1.00 (ref)	
18.5–23.9	0.64 (0.35–1.16)	0.138	0.53 (0.19–1.51)	0.237	0.58 (0.28–1.21)	0.149	0.42 (0.17–1.02)	0.054
24.0–27.9	0.28 (0.14–0.54)	< 0.001	0.21 (0.07–0.63)	0.006	0.28 (0.12–0.64)	0.003	0.29 (0.11–0.77)	0.013
≥ 28	0.40 (0.16–0.96)	0.041	0.16 (0.03–0.74)	0.019	0.56 (0.19–1.70)	0.305	0.28 (0.08–1.07)	0.063
WC (cm)	1.00 (0.98–1.03)	0.996	1.00 (0.96–1.04)	0.980	1.00 (0.97–1.04)	0.869	0.98 (0.95–1.02)	0.344

WHtR, waist to height ratio; BMI, body mass index; HR, hazard ratio; CI, confidence interval

### Propensity score match

**Fig. 2:**
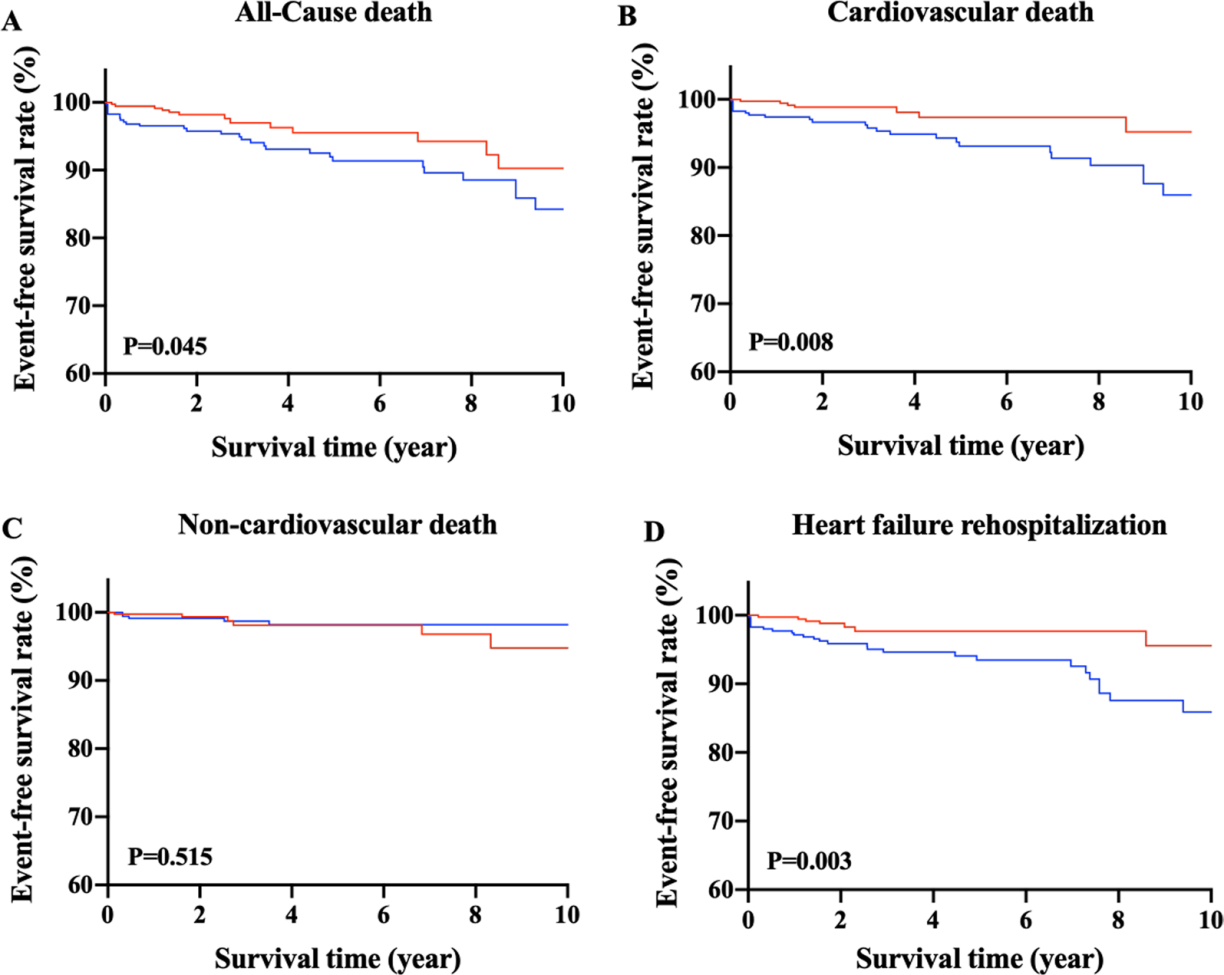
Kaplan–Meier survival curves for each outcome in propensity score-matched patients with low and high waist to height ratio. Event-free survival rates from (**A**) all-cause death, (**B**) cardiovascular death, (**C**) non-cardiovascular death, and (**D**) heart failure rehospitalization

Additional propensity score matching was performed to validate the association between WHtR and the risk of death in HFpEF patients. The propensity score-matched patients (N = 700) with low and high WHtR showed no significant difference of baseline characteristics (Additional file 2). Besides, patients with high WHtR had significantly higher risks of all-cause death (Fig. 2A), cardiovascular death (Fig. 2B), and HF rehospitalization (Fig. 2D), while the risks of non-cardiovascular death between two groups did not differ significantly (Fig. 2C).

### Subgroup analysis

**Fig. 3:**
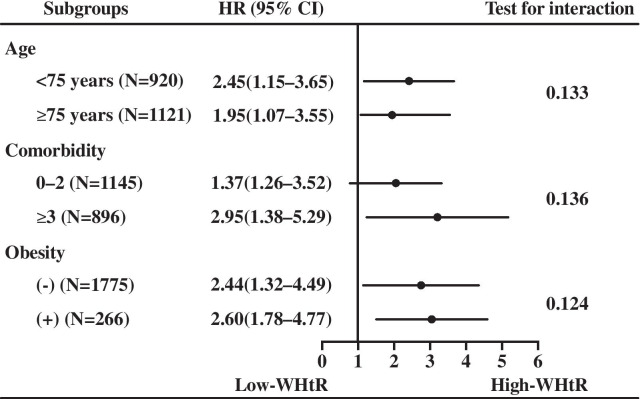
Association between waist to height ratio and all-cause death in the subgroups. WHtR, waist to height ratio. HR, hazard ratio; CI, confidence interval

We also explored the association between WHtR and all-cause death in different subgroup, as shown in Fig. 3. As the patients in this study were veterans and only a few females were included, thus we did not observe this association in gender subgroup. There were no significant interactions between WHtR and age, BMI, or obesity. The statistically significant association was not observed in each subgroup, however, subgroups with high WHtR exhibited higher risk of all-cause mortality than those with low WHtR.

## Discussion

This study of 2041 Chinese patients with HFpEF described the clinical characteristics of patients with low and high WHtR in detail, and comprehensively analyzed the associations between WHtR and all-cause death. The results demonstrated that: (1) abdominal obesity, overweight or obesity were highly prevalent in patients with HFpEF; (2) except HF, HFpEF patients had an average of 2–3 comorbidities, and those with high WHtR had heavier comorbidity burden than those with low WHtR; (3) HFpEF patients with high WHtR presented more significant left ventricular enlargement and hypertrophy as well as more severe diastolic dysfunction; (4) High WHtR was an independent risk factor for all-cause death in HFpEF patients, which was still observed in all subgroups.

Our HFpEF patients had lower prevalence of overweight or obesity [8, 24] while higher prevalence of abdominal obesity than HFpEF patients from other countries [8], which reflects different obesity patterns among patients with different races, and abdominal obesity deserves much more attention in Chinese patients with HFpEF. HFpEF is often accompanied by a variety of comorbidities [25, 26], which not only complicate clinical diagnosis and treatment, but also worsen prognosis and quality of life and increase hospitalization expenses [27]. In the present study, HFpEF patients had an average 2.38 comorbidities (excluding the 1 point assigned for HF), which was consistent with the results of the Spanish RICA registry study of heart failure [28]. In addition, patients with high WHtR had more comorbidities and also prescribed more drugs (ACEI/ARB, beta blocker and diuretic) than those with low WHtR, indicating that HFpEF is a multiorgan disease involving not only cardiac dysfunction but also noncardiac comorbidities contributing to clinical HF development [29], and abdominal obesity represented by WHtR may corelate to higher prevalence of multiple comorbidities in HFpEF patients.

Obesity have been demonstrated to exerted direct and indirect effects on the cardiovascular system, including increased myocardial load due to volume expansion, deterioration of arterial hypertension, left ventricular hypertrophy, and increased aortic stiffness [30]. In our study, HFpEF patients with high WHtR showed significantly higher levels of echocardiographic parameters, indicating that these patients have more significant left ventricular enlargement and hypertrophy, accompanied with more severe diastolic dysfunction. And this rising trend of echocardiographic parameters along with increasing WHtR was also in accordance with the elevated level of NT-proBNP (released in response to increased pressure or volume overload), which has been proved to predict HF events and mortality in a wide variety of HF cohorts [31].

HFpEF Patients probably have slightly better survival. However, with the increasing prevalence as time passes, its mortality remained unchanged, making HFpEF becoming the most common form of HF [32]. These trends emphasize the significance of studies to figure out the pathophysiology of HFpEF and establish effective therapeutic strategies against it. Sadly, there seems no effective treatments to improve the prognosis of HFpEF patients. HFpEF is characterized as the combination of multiple comorbidities, such as diabetes, obesity, chronic lung diseases, anemia, etc. Theses proinflammatory comorbidities interact and drive myocardial inflammation and fibrosis, oxidative stress, and the alteration in cardiomyocyte signaling pathways, which induce microvascular dysfunction and cardiomyocyte remodeling, and eventually left ventricular dysfunction [4, 33, 34]. Obesity, especially abdominal obesity, is a predominant comorbidity of HFpEF. It has played a crucial role in the incidence and development of HFpEF, thus understanding the impact of abdominal adiposity facilitates better exploring the pro-inflammatory pathology. As a positive endocrine organ, adipose tissue is able to produce multiple proinflammatory cytokines (e.g., tumor necrosis factor-alpha, interleukin-1, interleukin-18) that may cause diastolic dysfunction [35, 36]. Moreover, animal experiments have also indicated close associations between visceral obesity and increased cardiac macrophage infiltration and cytokine gene expression, aggravating myocardial hypertrophy, fibrosis and injury [37]. However, few literatures have investigated the impact of abdominal obesity on the mortality and HF deterioration in HFpEF patients. What’s more, although WC can reflect abdominal obesity in some extent, it might ignore a certain group of patients with short height and more adipose, and its diagnostic criterion for abdominal obesity varies with human race. Currently, WHtR as an indicator for abdominal obesity has proven to be a more accurate and advantageous screening tool than WC and BMI for identifying cardiovascular metabolic risk in adults [15, 38], because it avoids the need for age-, sex- and ethnic-specific boundary values in adults. 0.5 has been internationally recognized as the diagnostic threshold for WHtR [15, 39, 40]. In Chinese population, Zhang et al. conducted a survey of cardiovascular risk factors among approximately 35,000 people and found that the incidence of hypertension, dyslipidemia and hyperglycemia was significantly reduced with WHtR < 0.50 [41]. In this study, we used 0.5 as the threshold, finding that higher WHtR (≥ 0.5) was associated with higher risks of all-cause death, consistent with several previous studies [8, 42–44], and this association was also observed in propensity score-matched patients and all subgroups. Our findings suggests that abdominal obesity reflected by WHtR is associated with poor cardiovascular outcomes independent of BMI and WC. This association also holds true in non-obese individuals. Thus, both the amount and the distribution of adipose tissue may be important in patients with HFpEF. However, it should be noted that 95.84% of the patients in our study were male, thus our conclusion maybe more suitable for male patients. Besides, the threshold of 0.5 was derived from investigations mainly conducted among healthy populations not among patients with specific diseases, whether 0.5 could be a suitable boundary value for HFpEF patients lacks solid evidence and should be used carefully. For HFpEF patients, high WHtR may imply worsening outcomes, so more medication should be considered with priority for these patients. In addition, reducing abdominal obesity through diet, exercise, or both may be the fundamental and essential treatment for patients with HFpEF [45]. Because the long-term outcome of weight loss in HFpEF patients remains unclear, randomized controlled trials are needed to evaluate whether interventions to reduce abdominal obesity successfully reduce risk in HFpEF patients.

Additionally, we evaluated the relationship of BMI in different categories with adverse outcomes in HFpEF (Table 3). Patients with BMI < 18.5 kg/m^2^ were used as reference group. Although some HRs showed no statistical significance, we still observed the phenomenon of obesity paradox, as patients with BMI of 24–27.9 kg/m^2^ has the lowest HRs across each endpoint. That is, in our data, the overweight patients with HFpEF tended to have the lowest while those with BMI < 18.5 kg/m^2^ had the highest risks of adverse outcomes. Our results were different from the previous study [24], which demonstrated that the lowest mortality was seen with BMI of 26.5–35 kg/m^2^ and the highest mortality risk was seen with BMI < 23.5 and > 35 kg/m^2^. We consider that these differences may be mainly attributed to race differences, as Asians are more likely to have a higher percentage of body fat at lower BMI and WC than westerners [46].

A study of Wormser et al. [47] found that whether assessed alone or in combination, BMI, WC, and waist-to-hip ratio did not exhibit significant incremental predictive value for first-onset cardiovascular disease over traditional risk factors, suggesting that anthropometric parameters provide limited predictive information. It is worth mentioning that these analyses were restricted to individuals without a history of cardiovascular disease at the initial examination. While for individuals who have already been involved with one or more cardiovascular diseases, anthropometric parameters such as BMI and WHtR may exert certain prognostic value. In our study, HRs (95% CI) of WHtR regarding to all-cause death increased from 1.40 (0.89–2.21) of Model 1 to 1.91 (1.06–3.45) of Model 2, indicating that the existence of abdominal obesity (i.e., higher WHtR in our study) may accelerate the deterioration of diseases in HFpEF patients. However, whether WHtR can provide incremental prognostic information over traditional cardiovascular risk factors in HFpEF patients requires further investigations.

There are some limitations to this study. First, we had incomplete measurement of diastolic parameters. The data on mitral annular early diastolic velocity and left atrial volume index were absent and thus failed to be further analyzed. While other diastolic parameters, such as TR velocity (indicates functional alteration), LVMI and RWT (both indicate structural alterations) were available. TR velocity > 2.8 m/s, LVMI > 115/95 g/m^2^ (male/female), or RWT > 0.42 can be used as the evidence of abnormal morphology or diastolic dysfunction to help identify HFpEF. Secondly, it is a single-center study; however, to date there is no multicenter clinical study with a larger sample. Thirdly, HFrEF, HFpEF and HF with midrange ejection fraction have different clinical characteristics, pathophysiology, treatment and prognosis, which should be further verified in future studies.

## Conclusions

Our findings demonstrate that abdominal obesity is highly prevalent in HFpEF patients, as is the presence of multiple comorbidities. Higher WHtR is an independent risk factor for all-cause death in Chinese patients with HFpEF. Further studies are required to more comprehensively illuminate the mechanisms of the association between abdominal obesity and adverse outcomes in patients with HFpEF.

## Supplementary Information

**Additional files 1: Supplementary Table 1.** Cox regression analysis adjusted by covariates in Model 2.

**Additional files 2: Supplementary Table 2.** Baseline characteristics of propensity score-matched patients with low and high WHtR.

## Data Availability

The datasets used and/or analyzed during the current study are available from the corresponding author on reasonable request.

## Abbreviations

WHtR: Waist to height ratio
HFpEF: Heart failure with preserved ejection fraction
CCI: Charlson Comorbidity Index
LVEF: Left ventricular ejection fraction
ACEI: Angiotensin-converting enzyme inhibitors
ARB: Angiotensin receptor blockers
WC: Waist circumference
BMI: Body mass index
SBP: Systolic blood pressure
DBP: Diastolic blood pressure
HR: Heart rate
FG: Fasting glucose
eGFR: Estimated glomerular filtration rate
NT-proBNP: N-terminal pro-brain natriuretic peptide
IVS: Interventricular septal thickness
LVPWT: Left ventricular posterior wall thickness
LVEDD: Left ventricular end diastolic diameter
LVESD: Left ventricular end systolic diameter
LAD: Left atrial diameter
LVESV: Left ventricular end systolic volume
LVEDV: Left ventricular end diastolic volume
LVEF: Left ventricular ejection fraction
LVMI: Left ventricular mass index
RWT: Relative wall thickness
TR: Tricuspid regurgitation
