# Supplementary material for: Waist to height ratio is associated with an increased risk of mortality in Chinese patients with heart failure with preserved ejection fraction
Source: BMC Cardiovasc Disord. 2021 May 28;21:263. doi: 10.1186/s12872-021-02080-9 (PMC8164240; doi:10.1186/s12872-021-02080-9)
Supplement: Supplementary file 1 — Additional files 1: Supplementary Table 1. Cox regression analysis adjusted by covariates in Model 2. [file 12872_2021_2080_MOESM1_ESM.docx]

Supplementary Table 1. Cox regression analysis adjusted by covariates in Model 2

| Covariates | All-cause  death | | Cardiovascular  death | | Non-cardiovascular  death | | Heart failure rehospitalization | |
| --- | --- | --- | --- | --- | --- | --- | --- | --- |
|  | HR (95% CI) | *p* | HR (95% CI) | *p* | HR (95% CI) | *p* | HR (95% CI) | *p* |
| Age | 1.16(1.13–1.19) | <0.001 | 1.16(1.11–1.21) | <0.001 | 1.16(1.12–1.20) | <0.001 | 1.15(1.11–1.19) | <0.001 |
| Gender | 2.33(0.84–6.46) | 0.106 | 3.25(0.83–7.35) | 0.076 | 1.26(0.38–4.15) | 0.702 | 1.40(0.49–4.01) | 0.525 |
| SBP | 1.01(1.00–1.02) | 0.087 | 1.00(1.03–1.01) | 0.080 | 1.01(0.99–1.02) | 0.396 | 1.01(1.00–1.03) | 0.034 |
| DBP | 0.96(0.95–0.98) | <0.001 | 0.97(0.95–1.00) | 0.025 | 0.96(0.94–0.98) | <0.001 | 0.97(0.95–1.00) | 0.021 |
| Heart rate | 1.01(1.00–1.02) | 0.210 | 1.00(0.98–1.01) | 0.750 | 1.01(1.00–1.03) | 0.115 | 1.01(0.99–1.0) | 0.261 |
| Alcohol | 1.07(0.74–1.57) | 0.709 | 2.58(1.47–4.52) | 0.001 | 0.58(0.34–1.00) | 0.048 | 2.07(1.24–3.46) | 0.005 |
| Smoking | 1.84(1.34–2.53) | <0.001 | 1.06(0.64–1.77) | 0.822 | 2.42(1.58–3.71) | <0.001 | 1.18(0.74–1.87) | 0.492 |
| eGFR | 1.01(1.00–1.02) | 0.024 | 0.99(0.98–1.00) | 0.055 | 1.01(1.01–1.02) | <0.001 | 0.99(0.98–1.00) | 0.021 |
| NT-proBNP | 1.61(1.11–2.03) | <0.001 | 1.19(1.13–1.26) | <0.001 | 1.96(0.99–1.20) | 0.081 | 1.20(1.14–1.26) | <0.001 |
| Number of comorbidities | 1.29(1.16–1.44) | <0.001 | 1.36(1.15–1.61) | <0.001 | 1.25(1.08–1.44) | 0.002 | 1.23(1.06–1.44) | 0.007 |
| ACIE/ARB | 0.98(0.70–1.38) | 0.915 | 1.31(0.78–2.20) | 0.314 | 0.79(0.50–1.26) | 0.330 | 0.94(0.58–1.53) | 0.806 |
| Beta blocker | 0.90(0.64–1.27) | 0.560 | 1.00(0.60–1.67) | 0.990 | 0.81(0.52–1.27) | 0.364 | 1.24(0.78–1.98) | 0.362 |
| Diuretics | 0.93(0.59–1.48) | 0.765 | 0.62(0.28–1.40) | 0.249 | 1.05(0.58–1.88) | 0.876 | 1.16(0.63–2.16) | 0.629 |
| Statins | 0.82(0.58–1.15) | 0.244 | 0.55(0.33–0.91) | 0.020 | 1.10(0.69–1.77) | 0.686 | 0.68(0.42–1.10) | 0.119 |

SBP, systolic blood pressure; DBP, diastolic blood pressure; eGFR, estimated glomerular filtration rate; NT-proBNP, N-terminal pro-brain natriuretic peptide; ACEI, angiotensin-converting enzyme inhibitors; ARB, angiotensin receptor antagonist; HR, hazard ratio; CI, confidence interval.
