# Supplementary material for: Waist to height ratio is associated with an increased risk of mortality in Chinese patients with heart failure with preserved ejection fraction
Source: BMC Cardiovasc Disord. 2021 May 28;21:263. doi: 10.1186/s12872-021-02080-9 (PMC8164240; doi:10.1186/s12872-021-02080-9)
Supplement: Supplementary file 2 — Additional files 2: Supplementary Table 2. Baseline characteristics of propensity score-matched patients with low and high WHtR. [file 12872_2021_2080_MOESM2_ESM.docx]

Supplementary Table 2. Baseline characteristics of propensity score-matched patients with low and high WHtR

| Variable | Low-WHtR  (N=350) | High-WHtR  (N=350) | All  (N=700) | *p*-value |
| --- | --- | --- | --- | --- |
| Age (yrs) |  |  |  |  |
| <75 | 196(56.00) | 195(55.71) | 391(55.88) | 0.939 |
| ≥75 | 154(44.00) | 155(44.29) | 309(44.14) |  |
| Male (%) | 330(94.286) | 326(93.14) | 656(93.71) | 0.533 |
| Smoking (%) | 135(54.00) | 150(60.0) | 285(57.0) | 0.175 |
| Alcohol (%) | 127(50.80) | 141(56.4) | 268(53.6) | 0.209 |
| BMI (kg/m^2^) |  |  |  |  |
| <18.5 | 0 | 0 | 0 | - |
| 18.5-23.9 | 258(73.71) | 256(73.14) | 514(73.43) | 0.864 |
| 23.4-27.9 | 78(22.29) | 82(23.43) | 160(22.86) | 0.719 |
| >28 | 14(4.00) | 12(3.43) | 26(3.71) | 0.689 |
| Medication (%) |  |  |  |  |
| ACEI/ARB | 90(25.71) | 107(30.57) | 197(28.14) | 0.153 |
| Beta blocker | 77(22.00) | 75(21.61) | 152(21.81) | 0.902 |
| Diuretic | 30(8.57) | 41(11.82) | 71(10.14) | 0.168 |
| Statins | 222(63.43) | 220(63.40) | 442(63.42) | 0.994 |
| Comorbidities |  |  |  |  |
| ≤2 | 250(71.43) | 239(68.29) | 489(69.86) | 0.365 |
| 3-4 | 78(22.29) | 93(26.57) | 171(24.43) | 0.187 |
| ≥5 | 22(6.29) | 18(5.14) | 40(5.71) | 0.515 |

Data are presented as percentages. WHtR indicates waist to height ratio; BMI, body mass index; ACEI, angiotensin-converting enzyme inhibitors; ARB, angiotensin receptor antagonist.
